# High expression of nucleoporin 133 mRNA in bone marrow CD138+ cells is a poor prognostic factor in multiple myeloma

**DOI:** 10.18632/oncotarget.25350

**Published:** 2018-05-18

**Authors:** Soushi Ibata, Masayoshi Kobune, Shohei Kikuchi, Masahiro Yoshida, Shogo Miura, Hiroto Horiguchi, Kazuyuki Murase, Satoshi Iyama, Kohichi Takada, Koji Miyanishi, Junji Kato

**Affiliations:** ^1^ Department of Hematology, Sapporo Medical University School of Medicine, Sapporo, Japan; ^2^ Department of Medical Oncology, Sapporo Medical University School of Medicine, Sapporo, Japan

**Keywords:** multiple myeloma, NUP133, prognostic factor

## Abstract

Recent advances in plasma cell biology and molecularly-targeted therapy enable us to employ various types of drugs including immunomodulatory drugs, proteasome inhibitors, and immunotherapy. However, the optimal therapeutic strategies to introduce these drugs for heterogeneous patients with multiple myeloma (MM) have not yet been clarified. In the present study, we attempted to identify a new factor indicating poor prognosis in CD138+ myeloma cells using accumulated Gene Expression Omnibus (GEO) datasets from studies of MM and to assess the relationship between gene expression and survival using MAQC-II Project Myeloma (GSE24080). Five GEO datasets (GSE5900, GSE58133, GSE68871, GSE57317 and GSE16791) which were analyzed by the same microarray platform (GLP570) were combined into one MM database including various types of MM. However, we found that gene expression levels were quite heterogeneous. Hence, we focused on the differentially-expressed genes (DEGs) between newly-diagnosed MM and relapsed/refractory MM and found that the expression levels of more than 20 genes changed two-fold or more. Additionally, pathway analysis indicated that six pathways including Hippo signaling were significantly enriched. Then, we applied all DEGs and genes associated with core enrichment for GSE24080 to evaluate their involvement in disease prognosis. We found that nucleoporin 133 (NUP133) is an independent poor prognostic factor by Cox proportional hazard analysis. These results suggested that high expression of NUP133 could be useful when choosing the appropriate MM therapy and may be a new target of MM therapy.

## INTRODUCTION

Recently, several novel therapeutics including immunomodulatory reagents, proteasome inhibitors, histone deacetylase inhibitors (HDAC inhibitor), and monoclonal antibodies (mAbs) such as anti-CD38 and anti-SLAMF7 have been introduced to treat patients with multiple myeloma (MM) [1, 2]. However, the optimal therapeutic strategies to utilize these reagents have not yet been clarified. One plausible reason is that multiple myeloma (MM) is genetically heterogeneous and contains a variety of cytogenetic abnormalities such as del(17q), t(4;14) and t(14;16) [3–5]. Moreover, the condition of patients is heterogeneous probably because myeloma cells release not only M-protein but also several factors that sometimes induce advancing osteoporosis, amyloidosis and POEMS syndrome [6]. Hence, precise evaluation of patients with MM before treatment should be required to select the optimal therapeutic strategy.

Previously, the International Staging System (ISS) was used as a cost-effective staging system as well as the Durie-Salmon staging system [7]. The ISS is based on the assessment of two blood test results consisting of ß2-microglobulin (ß2M) and albumin. The combination of both serum biomarkers reflecting the condition of patients with MM has been shown to discriminate between three stages of myeloma and to be somewhat useful for identifying aggressive myeloma. However, in the last decade, it has been revealed that some chromosomal abnormalities detected by fluorescence *in situ* hybridization (FISH) are expressly involved in drug resistance to conventional chemotherapy. Thus, considering the cytogenetic risk, a revised International staging system (R-ISS) for MM has been developed and is now widely adopted [8, 9]. However, some of the novel reagents described above have been shown to overcome some cytogenetic risks. Therefore, much effort is focused on the identification of new prognostic factors associated with newly-diagnosed MM (NDMM) or relapsed/refractory MM (RRMM) [10–12].

Recently, a variety of types of data have been deposited in public databases. The data derived from microarray analysis have been compiled as a database in Gene Expression Omnibus (GEO) or ArrayExpress. Each database was usually utilized to confirm the reproducibility of our own data and sometimes to reprocess to obtain new results. To get an overview of these databases, some of them were analyzed according to similar sample sources, for example, CD138+ cells derived from bone marrow (BM). Moreover, such databases were often analyzed using an identical microarray or platform. Some datasets were analyzed regarding BM CD138+ cells derived from healthy volunteers (HV) or NDMM, while others were analyzed regarding BM CD138+ cells derived from smoldering MM (SMM) or NDMM. When the platform was identical, combined use of these datasets should be readily achieved.

Here, we first attempted to identify datasets regarding CD138+ cells from GEO and accumulate the data into one large dataset. After normalization, we analyzed differentially-expression genes (DEGs) and significantly altered pathways in RRMM as compared with NDMM. Moreover, we assessed the relationship between genes identified by these processes and survival by using the publicly accessible MAQC-II Project MM dataset (GSE24080).

## RESULTS

### Confirmation of data accumulation in myeloma dataset #1 and #2

In an attempt to combine the datasets involving CD138+ plasma cells, five datasets analyzed using an identical array (platform) were combined into one myeloma dataset #1. After normalization, DEGs (HV vs. plasma cell dysplasia) were analyzed and all DEGs were visualized as a heatmap to give an overview of the change in expression of genes during disease progression (Supplementary Figure 1). The results showed that gene expression was quite heterogeneous, especially in monoclonal gammopathy of undetermined significance (MGUS) and SMM. We therefore decided to extract NDMM and RRMM to constitute myeloma dataset #2. Subsequently, all DEGs between NDMM and RRMM were selected and visualized as a heatmap (Supplementary Figure 2). This result suggested that gene expression could differ remarkably between NDMM and RRMM. Thus, we utilized myeloma dataset #2 for subsequent experiments.

### The results of DEGs and GSEA in RRMM

**Figure 1:**
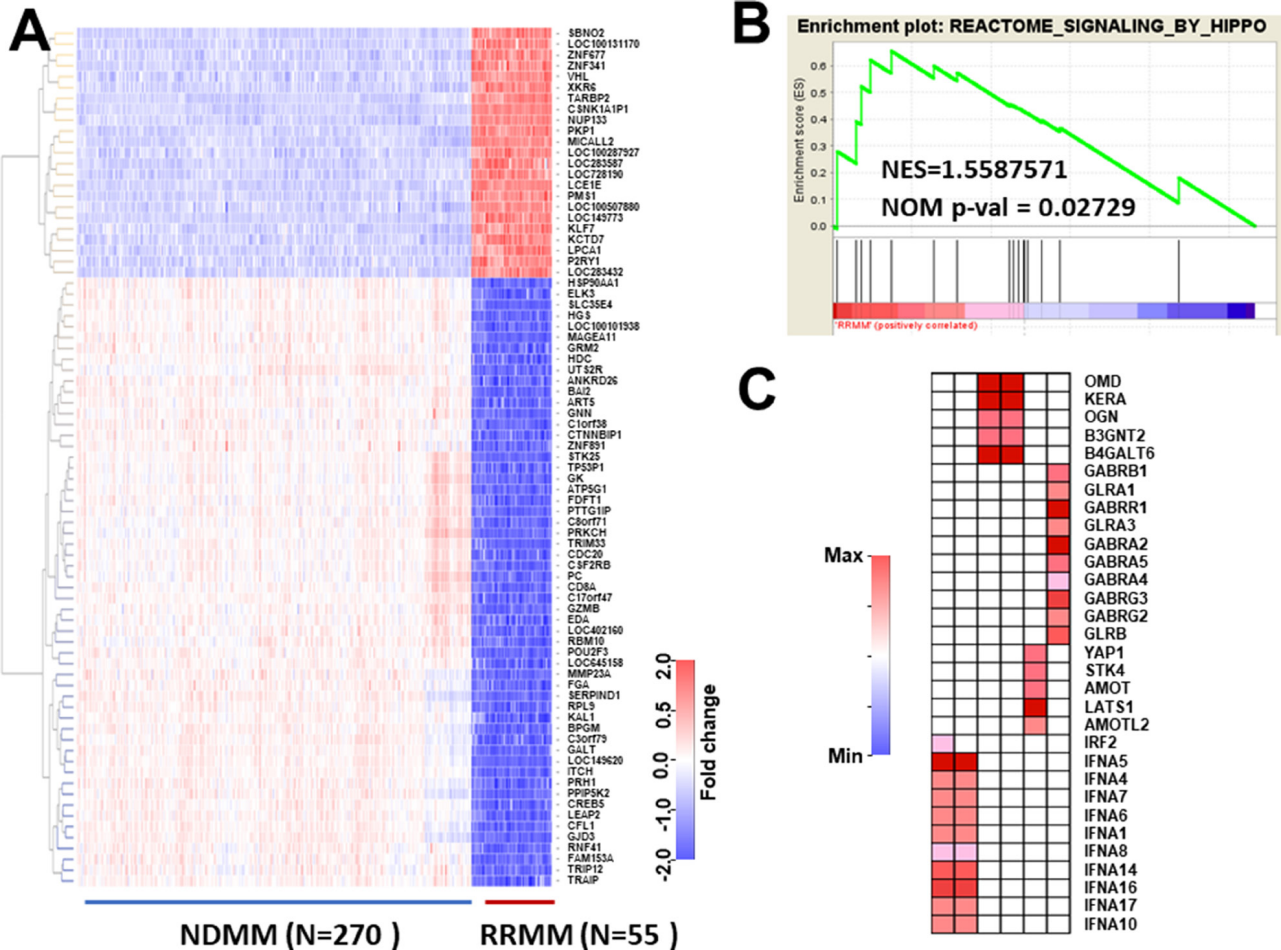
Selection of differentially-expressed genes (DEGs) and enriched pathways in RRMM (**A**) NDMM (*N* = 270) and RRMM (*N* = 55) were selected from whole myeloma large datasets by dyer package. Since this dataset was relatively large, bioinformatic significance was considered according to Bonferroni's method (*P* < 10 × 10^−148^). Additionally, genes showing more than a 2-fold change were selected and visualized as a heatmap. (**B**) The pathways associated with RRMM were analyzed by gene set enrichment analysis version 6. A NOM *P*-value < 0.05 was considered to be significant (Table 3). (**C**) Leading edge analysis was conducted by GSAE application. Genes showing core enrichment from six pathways were selected and visualized as a heatmap.

To analyze myeloma dataset #2, we first identified DEGs showing a more than two-fold change between NDMM and RRMM by analysis using limma package. To avoid selecting false negatives, we used quite a low *P*-value (*P* = 10^–148^) and a low *q*-value (*q* = 10^–70^) between DEGs in NDMM and RRMM as compared with the Bonferroni method (*P*-value = 6.574622 × 10^–6^). The results were visualized as a heatmap (Figure 1A) and showed that DEGs clearly discriminated between NDMM and RRMM. After confirmation, we focused on highly-increased DEGs which are listed in Table 1 because highly-increased DEGs could be potential biomarkers which could be analyzed by immunohistochemical staining or liquid biopsy [13] and may also be potential new molecular targets. Moreover, it is possible that some clones expressing DEGs in NDMM may contribute to clonal evolution and development into RRMM. The expression of CSNK1A1P1 (Casein Kinase 1 Alpha 1 Pseudogene 1) which encodes a long non-coding RNA was found to be elevated around 15.5-fold (15.5 = 2^3.955^) in RRMM. This gene could thus be a marker of RRMM. We next conducted enrichment analysis and found that six pathways were significantly enriched (*p <* 0.05, but FDR > 0.25) (Table 2). Among them, Hippo signaling has been reported to be involved in the development of MM (Figure 1B). We further assessed the genes associated with core enrichment by leading edge analysis using the GSEA web tool. Multiple genes such as STK4 and YAP1 involved in Hippo signaling were picked up (Figure 1C). Thus, analysis of DEGs and pathways in RRMM as compared with NDMM provided multiple candidate genes. However, whether these candidates are involved in the prognosis of MM was unclear. Hence, we employed another database to explore the prognostic factors from among candidate genes.

**Table 1:** Genes with expression highly elevated in RRMM compared with NDMM

Gene symbol	logFC	AveExpr	*t*	*P*-Value	*q*-Value
CSNK1A1P1	3.955	8.115	90.75	7.95E-236	5.56E-234
LOC283432	3.181	6.028	51.16	3.53E-159	5.37E-159
LOC100507880	3.136	8.625	48.80	3.35E-153	3.45E-153
TARBP2	2.979	8.676	67.02	1.54E-194	9.79E-194
NUP133	2.797	7.786	65.50	1.80E-191	9.71E-191
KLK7	2.797	5.373	49.27	2.16E-154	2.44E-154
MICALL2	2.710	7.799	69.14	1.01E-198	8.87E-198
LOC728190	2.603	5.970	54.12	2.11E-166	3.88E-166
LOC149773	2.423	4.810	64.68	8.59E-190	4.30E-189
SBNO2	2.350	5.761	61.83	8.02E-184	3.51E-183

All databases were normalized by the rma method and differentially-expressed genes (DEGs) were detected using the limma package. The average expression (AveExpr) column is the average of all arrays for all groups, not for one group. The *q*-value correlated with false discovery rate, which is the *P*-value adjusted for multiple comparisons. FC, fold change; logFC, log2FC.

**Table 2:** Summary of the results of GSEA analysis

Pathway (REACTOME)	SIZE	NES	NOM *P*-val
1. SIGNALING_BY_HIPPO	16	1.5587571	0.027290449
2. REGULATION_OF_IFNA_SIGNALING	24	1.5417559	0.025477707
3. LIGAND_GATED_ION_CHANNEL_TRANSPORT	20	1.5310737	0.014403292
4. KERATAN_SULFATE_KERATIN_METABOLISM	26	1.5274918	0.029045643
5. KERATAN_SULFATE_ BIOSYNTHESIS	22	1.4906782	0.04158004
6. TRAF6_MEDIATED_IRF7_ACTIVATION	27	1.4500268	0.04477612

GSAE analysis was conducted using gene sets derived from the Reactome pathway database (Reactome gene sets) in the Molecular Signatures Database v6.1.

### Identification of a new poor prognostic factor in patients with MM

To assess the relationship between candidate genes and survival, we used another dataset, The MicroArray Quality Control (MAQC)-II Project MM dataset (GSE24080) (www.ncbi.nlm.nih.gov/geo/query/acc.cgi?acc=GSE24080) [14]. In this dataset, gene expression profiling of highly-purified bone marrow plasma cells in NDMM (*N* = 559) was available. Moreover, this dataset was analyzed using an Affymetrix Human Genome U133 Plus 2.0 Array by which myeloma dataset #2 was also analyzed. Furthermore, supplementary data regarding clinical information such as survival time, the total number of deaths (*N* = 172), recurrence or progression (*N* = 249) and cytogenetic abnormalities of individual patients was included with this dataset. In this retrospective analysis, all CD138+BM cells derived from patients who followed up at the end of study was analyzed and average observation period was 49.9 ± 24.6 months. Hence, all candidate genes from myeloma dataset #2 could be readily analyzed regarding overall survival (OS) and event-free survival (EFS) on this dataset. After all data of GSE24080 were downloaded and combined with clinical information, the relationship between all candidate genes and OS was analyzed using the Cox proportional hazard model. The results are summarized in Table 3. Unexpectedly, most DEGs and genes associated with core enrichment pathways were not involved in OS. Notably, CSNK1A1P1, a highly increased DEG (Table 1) was not associated with OS, indicating that DEGs in RRMM are not always associated with the prognosis of NDMM. Fortunately, four candidate genes including GABRG2 (Gamma-Aminobutyric Acid Type A Receptor Gamma2 Subunit), GLRA1 (Glycine Receptor Alpha 1), IFNA4 (Interferon Alpha 4) and nucleoporin (NUP) 133 were shown to be significantly associated with poor OS. It is well known that cytogenetic abnormalities such as del(17q), t(4;14) and t(14;16) are associated with poor prognosis of NDMM. In fact, The MicroArray Quality Control (MAQC)-II Project MM dataset revealed that any cytogenetic abnormality (Cyto.Abn) was a significant poor prognostic factor in NDMM (data not shown). Therefore, we conducted a multivariate analysis in combination with Cyto.Abn using the Cox proportional hazard model [15]. The results revealed that NUP133 and Cyto.Abn could be regarded as independent prognostic factors (Table 4). These results indicated that high expression of NUP133 observed in RRMM was also detected in NDMM patients who showed poor OS.

**Table 3:** Univariate analysis of the relationship between gene expression and OS by Cox proportional hazard model using the publicly accessible MAQC-II Project MM dataset (GSE24080)

Gene symbol	Hazard ratio (95% CI)	*P* value
CSNK1A1P1	1.118 (0.943–1.325)	0.2001
GABRA2	0.934 (0.725–1.204)	0.5977
GABRA4	1.048 (0.890–1.235)	0.5715
GABRA5	1.037 (0.925–1.163)	0.5300
GABRB1	0.975 (0.841–1.130)	0.7314
**GABRG2**	**1.242 (1.048–1.471)**	0.0125^*^
GABRG3	1.045 (0.830–1.315)	0.7108
GABRR1	0.892 (0.726–1.095)	0.2731
**GLRA1**	**1.208 (1.011–1.444)**	0.0374^*^
GLRA3	0.985 (0.817–1.187)	0.8699
GLRB	0.978 (0.800–1.194)	0.8244
IFNA1	1.102 (0.973–1.249)	0.1249
**IFNA4**	**1.465 (1.076–1.997)**	0.0155^*^
IFNA5	0.909 (0.692–1.195)	0.4947
IFNA6	0.953 (0.836–1.086)	0.4658
IFNA7	0.971 (0.834–1.130)	0.7016
IFNA8	0.996 (0.886–1.119)	0.9459
IFNA10	1.120 (0.900–1.393)	0.3097
IFNA14	0.894 (0.661–1.209)	0.4677
IFNA16	0.758 (0.545–1.053)	0.0986
IFNA17	0.876 (0.598–1.282)	0.4946
IRF2	0.816 (0.597–1.114)	0.1995
KCTD7	1.019 (0.770–1.347)	0.8973
KERA	1.032 (0.834–1.276)	0.7731
KLK7	1.059 (0.812–1.380)	0.6744
LATS1	1.123 (0.872–1.445)	0.3700
LOC149773	1.040 (0.820–1.320)	0.7446
LOC283432	0.970 (0.878–1.072)	0.5534
LOC283587	1.087 (0.976–1.211)	0.1284
LOC728190	1.010 (0.709–1.438)	0.9567
LOC100287927	0.794 (0.593–1.062)	0.1204
LOC100507880	0.949 (0.786–1.146)	0.5893
LPCAT1	0.931 (0.800–1.083)	0.3523
MICALL2	0.850 (0.652–1.107)	0.2270
**NUP133**	**1.658 (1.150–2.392)**	0.0068^*^
OGN	1.139 (0.944–1.375)	0.1734
OMD	1.139 (0.920–1.411)	0.2325
P2RY1	0.920 (0.791–1.069)	0.2753
PATID	0.999 (0.999–1.000)	0.0581
PKP1	1.013 (0.784–1.310)	0.9205
SBNO2	0.988 (0.811–1.204)	0.9056
STK4	1.123 (0.764–1.650)	0.5561
TARBP2	1.101 (0.708–1.712)	0.6705
XKR6	0.859 (0.656–1.124)	0.2679
YAP1	0.820 (0.621–1.083)	0.1621
ZNF677	0.966 (0.778–1.199)	0.7529

Significant genes associated with OS were indicated in Bold font. ^*^P < 0.05.

**Table 4:** Multivariate analysis by the Cox proportional hazard model with stepwise regression using the Bayesian information criterion (BIC)

Gene symbol	Hazard ratio (95% CI)		*P*-value
NUP133	1.55	(1.14–2.09)	4.7E-03
Cyto.Abn	2.15	(1.58–2.92)	9.1E-07

Cyto.Abn, Cytogenetic abnormality including chromosomal abnormality, translocation and deletion detected by fluorescence *in situ* hybridization. Concordance probability = 0.644.

### Analysis of OS and EFS in patients with CD138+ myeloma cells

**Figure 2:**
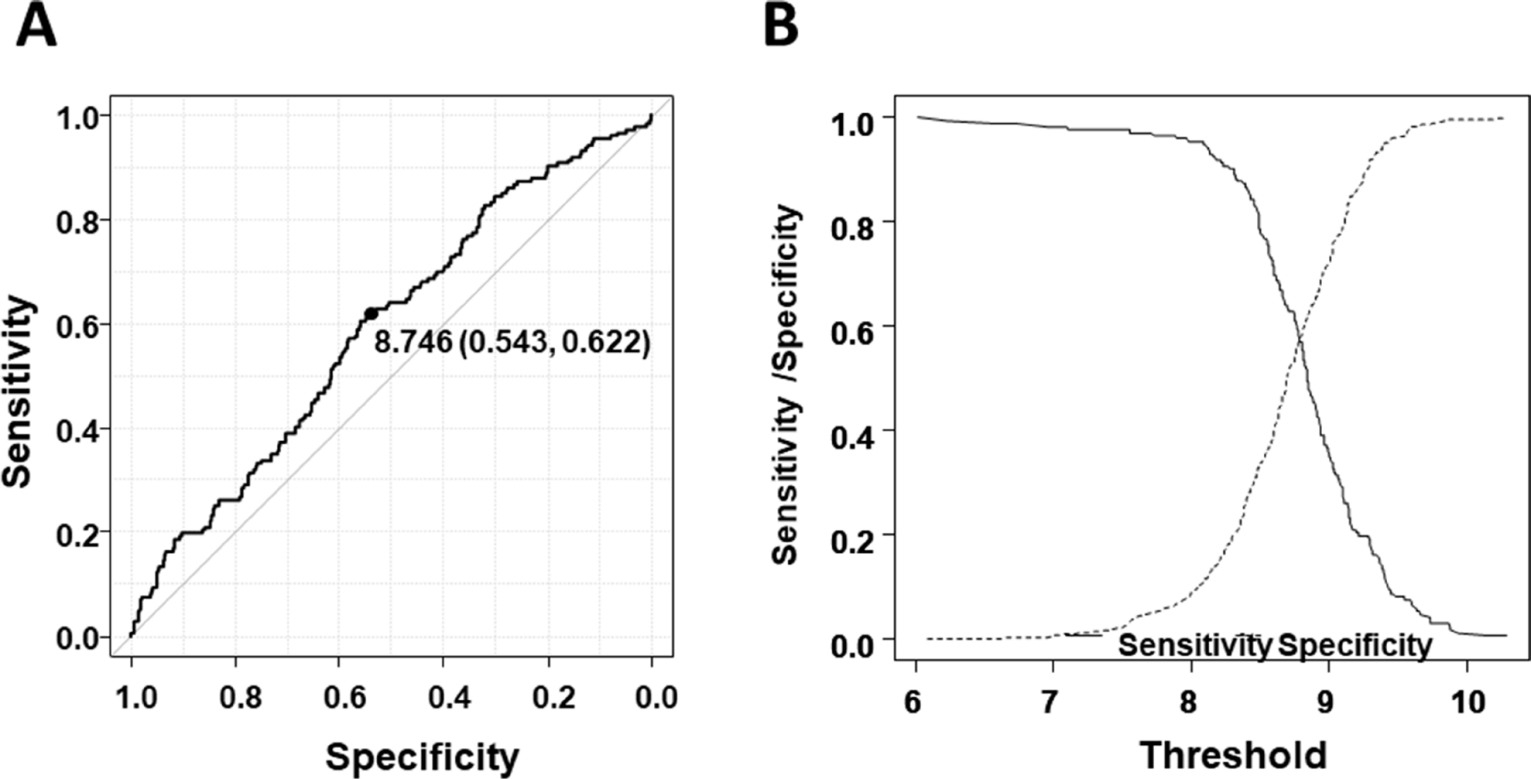
Determination of the cut-off level of NUP133 expression (**A**) ROC curve to determine the cut-off level of NUP133 associated with OS. Area under the curve: 0.5899 (95% CI: 0.5393–0.6405). (**B**) Alternative representation of threshold (cut-off level) resulting from minimal value of BER (Balanced Error Rate: Sensitivity/Specificity).

**Figure 3:**
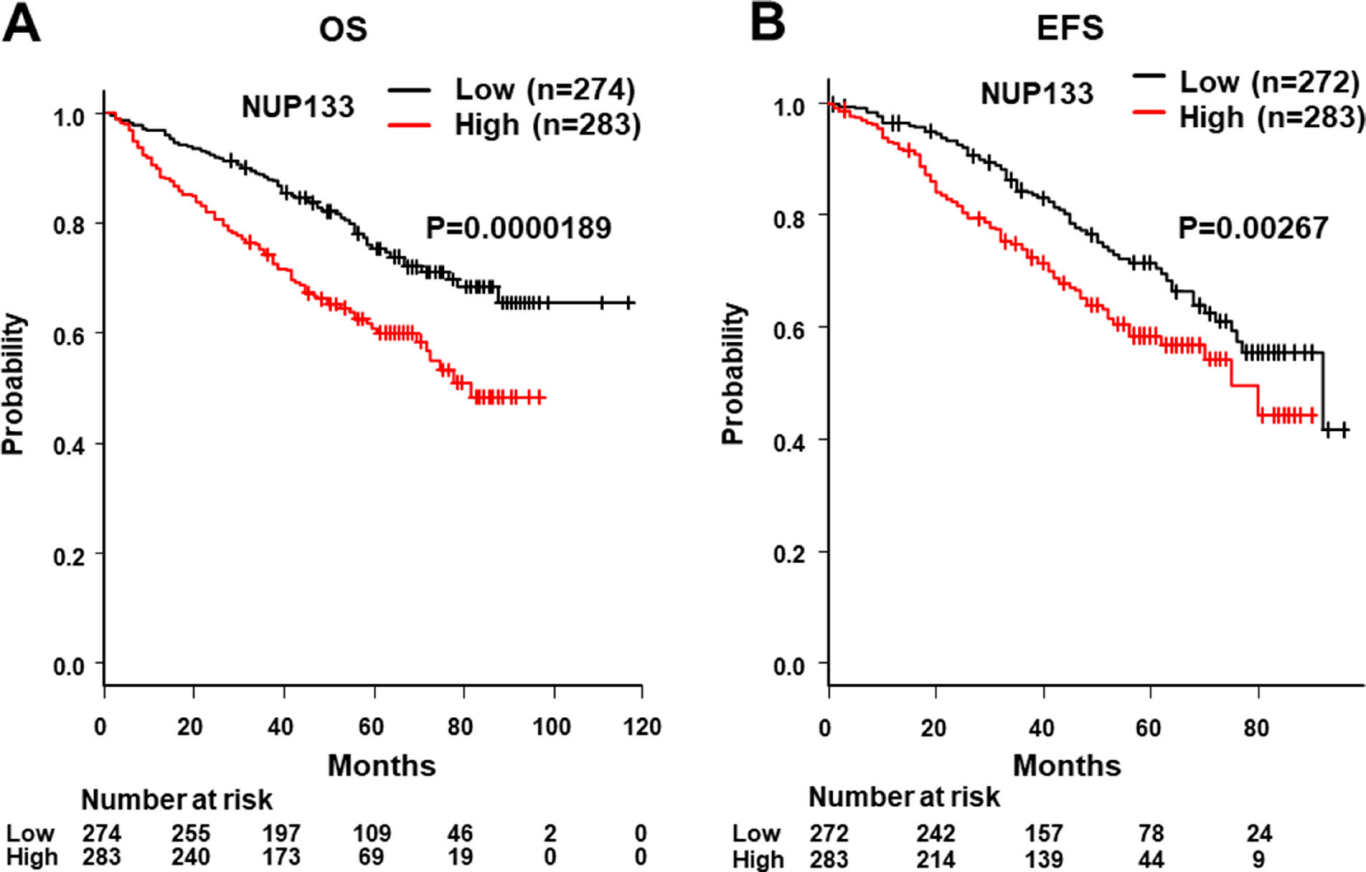
Analysis of OS and event-free survival (EFS) with high and low levels of NUP133 mRNA expression in CD138+ myeloma cells (**A**) Analysis of the relationship between OS and NUP133 expression in patients with myeloma using the publicly-accessible MAQC-II Project MM dataset (GSE24080) from the GEO. The high NUP133 expression group consisted of 284 patients with MM. The low NUP133 expression group consisted of 275 patients with MM. The median OS time of the high NUP133 group did not reach 50% (NA: not applicable). The median OS time of the low NUP133 group was 81 months (95% CI:71-NA). (**B**) The relationship between EFS and NUP133 expression in patients with myeloma was analyzed using the publicly-accessible MAQC-II Project MM database. The median EFS time of the high NUP133 group was 92 months (95% CI:75-NA). The median EFS time of the low NUP133 group was 75 months (95% CI:75-NA). The high and low NUP133 groups were divided by the result of the ROC curve as indicated in Figure 2.

We next determined the cut-off level of NUP133 and analyzed OS and EFS. To determine the cut-off level, we utilized ROC analysis using two-year OS. As shown in Figure 2, the cut-off level of NUP133 was 8.746 (Specificity = 0.543, sensitivity = 0.622) which was slightly higher than average expression (AveExpr) shown in Table 1. By using this cut-off level, patients with NDMM in the MAQC-II Project MM dataset were divided into two groups, high and low NUP133 expression. OS and EFS in the two groups were analyzed by log-rank test. As shown in Figure 3, the high NUP133 expression group exhibited significantly poorer prognosis not only with regard to OS, but also EFS. These results indicated that high expression of NUP133 predicted poor prognosis in NDMM.

## DISCUSSION

In the present study, we created a relatively large dataset by combining five datasets analyzing RNA expression of CD138+ cells. We then analyzed highly-expressed genes in RRMM. Moreover, we investigated whether these genes could predict poor prognosis of NDMM by using the MAQC-II Project MM dataset. We found that high expression of NUP133 could be an independent poor prognostic factor in NDMM.

NUP133 is one of the NUP family which consist of approximately 30 proteins. NUP proteins are the main components of the nuclear pore complex (NPC) which form large molecular channels across the nuclear envelope [16]. It has been shown that NPCs are involved in diverse functions including facilitating nucleocytoplasmic transport, as well as chromatin organization, the regulation of gene expression and DNA repair [17]. NUP family proteins can be roughly categorized into scaffold NUPs, embedded in the double membrane of the nuclear envelope, and FG-NUPs, FG (Phe and Gly)-repeat-containing-NUPs, which constitute the permeability barrier of the pore [16] and interact with the soluble nucleocytoplasmic transport machinery such as importins and exportins [18]. NUP133 is an essential scaffold NUP and disruption of the NUP133 gene results in clustering of NUPs. Thus, NUP133 should be involved in maintaining the position of the NPC within the nuclear envelope [19]. Additionally, NUP133 plays a critical role in mRNA export [20, 21].

It has been shown that some NUPs including NUP88, NUP98 and NUP214 make mechanistic contributions to carcinogenesis, particularly in leukemias [22]. Hence, we assessed the relationship between their expression levels and OS by using the MAQC-II Project MM dataset. However, we found no correlation between the expression level of these NUPs and OS in NDMM (Supplementary Table 1). Among NUP family members, it remains unclear how NUP133 alone could be involved in OS and EFS in NDMM.

In this regard, it has been reported that the N-terminal domain of NUP133 is required for efficient anchoring of the dynein/dynactin complex to the nuclear envelope in prophase through an interaction network via centromere protein F (CENP-F) and NudE/NudEL [23]. CENP-F is known to be associated with the centromere-kinetochore complex and NudE/NudEL is known to interact with dynein [24]. These findings suggest that NUP133 plays an important role in mitosis and chromosome partitioning. Further studies including knockdown and overexpression of NUP133 in myeloma cells will be required to clarify the role of NUP133 in MM.

In conclusion, the expression level of NUP133, a component of NPCs, could be involved in the prognosis of MM. These findings may be useful for understanding the development of MM and may provide a new therapeutic approach in MM.

## MATERIALS AND METHODS

### Analysis and data mining of public data base GEO (gene expression omnibus)

GEO datasets [GSE5900 (Healthy volunteer (HV): *N* = 22; Monoclonal gammopathy of undetermined significance (MGUS): *N* = 44; smoldering MM (SMM): *N* = 12), GSE58133 (newly-diagnosed MM (NDMM): *N* = 120), GSE68871 (NDMM: *N* = 118), GSE57317 (relapsed/refractory MM (RRMM): *N* = 55) and GSE16791 (NDMM: *N* = 32)] which were analyzed using the same gene chip (Affymetrix Human Genome U133 Plus 2.0 Array: Platform GLP570) were downloaded as separate CEL files. CEL data were background corrected using the Robust Multi-Array Average (RMA) or Microarray Suite 5.0 (MAS5) provided from analysis by Bioconductor (R commander 3.4.1).

Each data-set was saved as a matrix in text format and each dataset was reloaded into RStudio (version1.0.153) using the read table function. All the datasets were combined into one myeloma dataset using the cbind function provided by R commander. To normalize each dataset combined, quintile normalization was conducted using the affy Bioconductor package for this dataset and saved as a matrix in text format (myeloma dataset #1).

To select only the NDMM (*N* = 270) and RRMM (*N* = 55) from myeloma dataset #1, the dplyr package was utilized and the results were saved as another matrix in text format (myeloma dataset #2). A dendrogram and heatmap were created using amap, gplots and 3D heatmap packages.

To detect DEGs, limma package was used after RMA normalization (Table 1) and EdgeR package was used after MAS5 normalization (Supplementary Table 2).

### Gene set enrichment analysis and network analysis

Gene set enrichment analysis (GSEA) was conducted using the open source software GSEA 3.0 http://software.broadinstitute.org/gsea/index.jsp. For gene sets databases, h.all.v6.0.symbol.gmt [Hallmarks], c2.cp.biocarta.v6.0.symbols.gmt [Curated], c2.cp.kegg.v6.0.symbols.gmt, c2.cp.reactome.v6.0.symbols.gmt [Curated] and c6all.v6.0.symbols.gmt [Oncogenic signature] were used. The pathways showing NOM *P*-val (*P*-value) < 0.05 or false discovery rate (FDR) *q*-val (FDR) < 0.25 were considered as significant.

### Statistical analysis

Each dataset was first evaluated for normality of distribution by the Kolmogorov-Smirnov test to decide whether a non-parametric rank-based analysis or a parametric analysis should be used. The significance of differences between groups was assessed by one-way ANOVA with the post-hoc Tukey Honestly Significant Difference Test. Results are expressed as the mean ± standard deviation (SD). The significance of differences was assessed by either Student's *t*-test or the Mann-Whitney *U*-test, and a *P*-value < 0.05 was considered statistically significant. All statistical analyses were performed with R commander (version 3.4.1) or EZR (Easy R), which is a graphical user interface for R [25].

The relationship between DEGs and OS were evaluated using the publicly accessible MAQC-II Project MM dataset (GSE24080) from the GEO using the Cox proportional hazard model provided from EZR [26]. For a multivariate model, candidate genes (*P* value < 0.2) were selected because correlations can play an important role to build better prognostic models according to previous report [15].

## SUPPLEMENTARY MATERIALS FIGURES AND TABLES


