# Supplementary material for: High expression of nucleoporin 133 mRNA in bone marrow CD138+ cells is a poor prognostic factor in multiple myeloma
Source: Oncotarget. 2018 May 18;9(38):25127–35. doi: 10.18632/oncotarget.25350 (PMC5982762; doi:10.18632/oncotarget.25350)
Supplement: Supplementary file 1 [file oncotarget-09-25127-s001.pdf]

High expression of nucleoporin 133 mRNA in bone marrow CD138+ cells is a poor prognostic factor in multiple myeloma

SUPPLEMENTARY MATERIALS

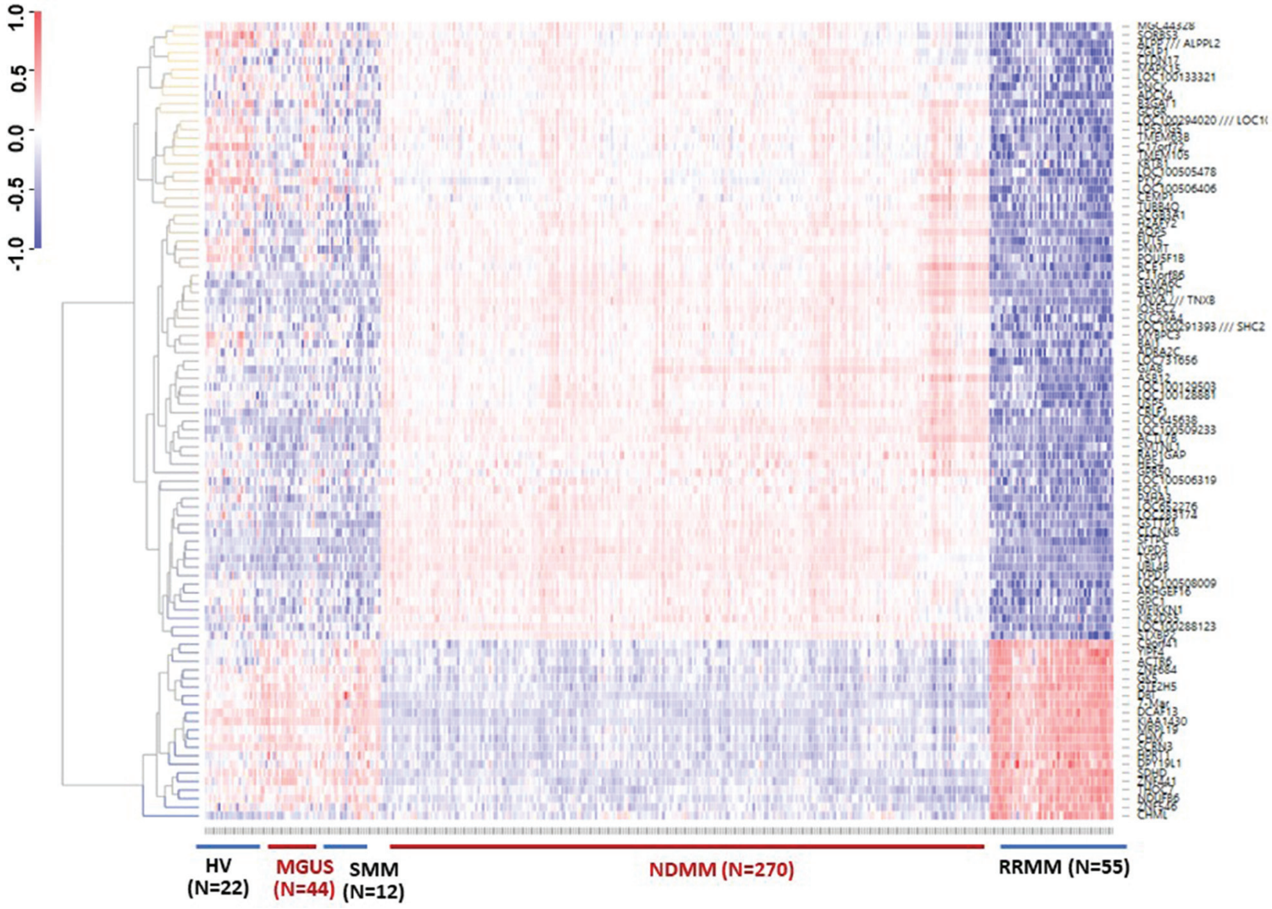

Supplementary Figure 1: Overview of gene expression in HV, MGUS, SMM, NDMM and RRMM. DEGs (differentially-expressed genes) were selected by a genefilter package when there was a significant difference (P-value $< 1 \times 10^{-16}$) between HV and plasma cell dysplasia (MGUS, SMM, NDMM and RRMM). Matrix data obtained was visualized by a 3D heatmap package.

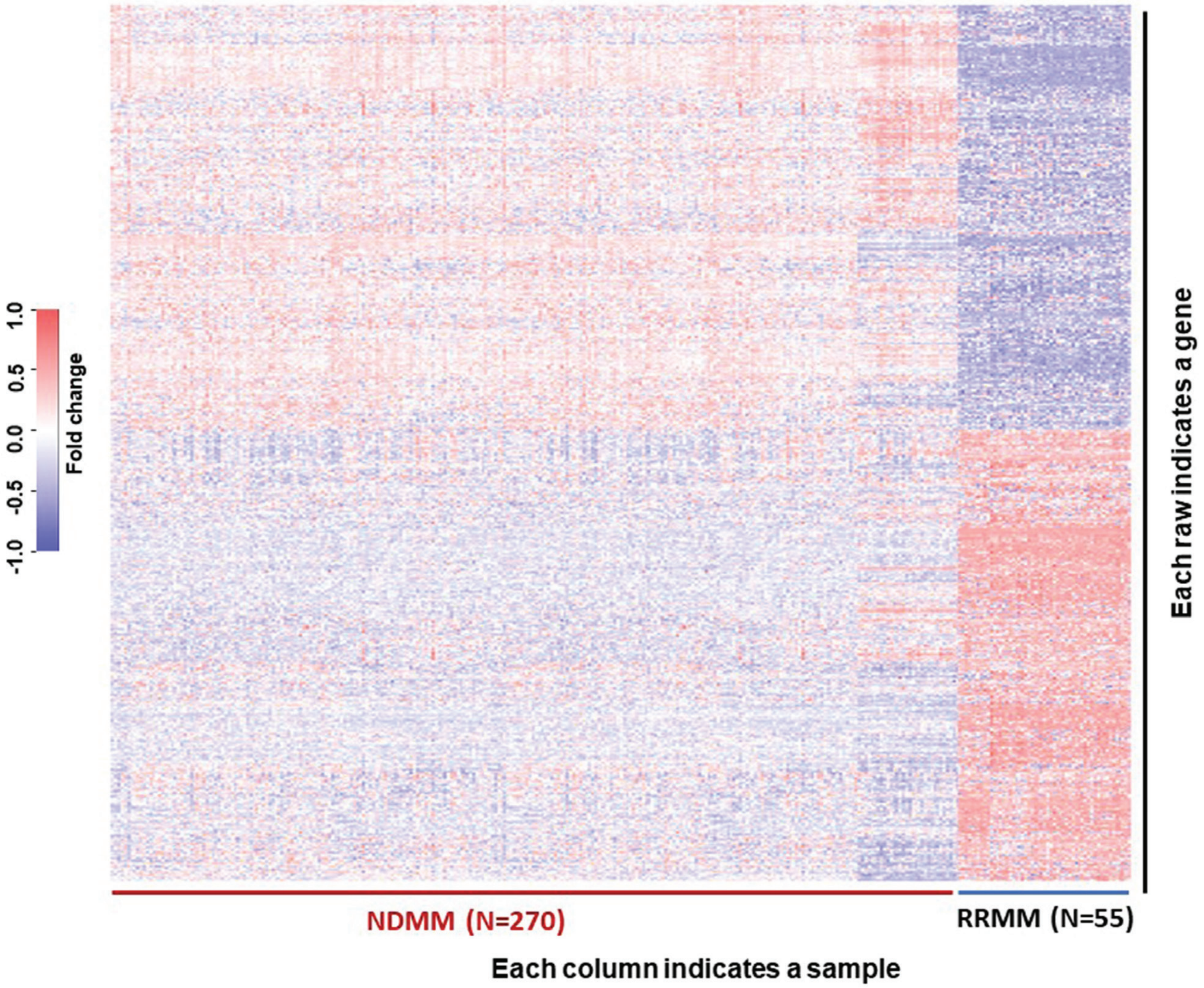

Supplementary Figure 2: All significant DEGs were visualized as a heatmap. DEGs between NDMM and RRMM were selected using a gene filter package. Since data size seems to be large, the significance of *P*-values was calculated by the Bonferroni method and determined as a *P*-value = 6.574622×10^{-6}. Many DEGs were detected but their gene expression level was found to be variable in both NDMM and RRMM.

Supplementary Table 1: Univariate analysis of the relationship between other NUPs and OS

Nucleoporin complex	Hazard ratio (95% CI)	*P*-value
NUP88	0.9427 (0.6693–1.328)	0.7355
NUP98	0.9588 (0.7174–1.281)	0.7759
NUP133	1.774 (1.3000–2.422)	0.0003
NUP214	0.7759 (0.4982–1.208)	0.2614

Supplementary Table 2: Genes with expression highly elevated in RRMM compared with NDMM

Gene symbol	logFC	*P*-Value	*q*-Value	*ranking_edgeR_ari*
CD53	4.342789644	1.60E-170	3.53E-169	517
**CSNK1A1P1**	**3.906767508**	**0.00E-00**	**0.00E-00**	**50.5**
CCS	3.624226167	4.31E-73	2.48E-72	1979
CCDC116	3.437426799	2.92E-82	1.93E-81	1721
**LOC283432**	**3.208728725**	**0.00E-00**	**0.00E-00**	**50.5**
FANCG	3.120939721	6.50E-84	4.42E-83	1674
AQP1	3.120308099	0.00E-00	0.00E-00	50.5
**LOC100507880**	**3.072585153**	**0.00E-00**	**0.00E-00**	**50.5**
KLHL4	2.992107564	7.65E-239	3.78E-237	230
GPR115	2.973494161	0.00E-00	0.00E-00	50.5
**TARBP2**	**2.963374094**	**0.00E-00**	**0.00E-00**	**50.5**
LOC151438	2.926591151	4.05E-241	2.06E-239	224
FBXO18	2.900921088	0.00E-00	0.00E-00	50.5
IGLC7	2.887737961	0.00E-00	0.00E-00	50.5
LOC286135	2.88407777	5.59E-137	8.12E-136	784
P2RY1	2.832604364	0.00E-00	0.00E-00	50.5
GNA13	2.83000744	0.00E-00	0.00E-00	50.5
**KLK7**	**2.811581605**	**0.00E-00**	**0.00E-00**	**50.5**
DHH	2.782843815	4.07E-260	2.41E-258	192
HIST1H2BG	2.779485041	4.17E-223	1.67E-221	284
TRIM25	2.764384632	6.41E-212	2.25E-210	324
HIF1A	2.759719059	1.45E-173	3.32E-172	496
**NUP133**	**2.75932369**	**0.00E-00**	**0.00E-00**	**50.5**
CUL7	2.733937567	2.16E-213	7.82E-212	314

All databases were normalized by the MAS5 method and differentially-expressed genes (DEGs) were detected using the EdgeR package. The *q*-value is the *P*-value adjusted for multiple comparisons, which correlate with false discovery rate. FC, fold change; logFC, log2FC. DEGs shown in Table 1 were indicated in Bold font.
